# Microfluidics Used as a Tool to Understand and Optimize Membrane Filtration Processes

**DOI:** 10.3390/membranes10110316

**Published:** 2020-10-29

**Authors:** Izabella Bouhid de Aguiar, Karin Schroën

**Affiliations:** Membrane Science and Technology—Membrane Processes for Food, University of Twente, P.O. Box 217, 7500 AE Enschede, The Netherlands; c.g.p.h.schroen@utwente.nl

**Keywords:** microfluidics, membrane, filtration processes, pore blocking, pore design

## Abstract

Membrane filtration processes are best known for their application in the water, oil, and gas sectors, but also in food production they play an eminent role. Filtration processes are known to suffer from a decrease in efficiency in time due to e.g., particle deposition, also known as fouling and pore blocking. Although these processes are not very well understood at a small scale, smart engineering approaches have been used to keep membrane processes running. Microfluidic devices have been increasingly applied to study membrane filtration processes and accommodate observation and understanding of the filtration process at different scales, from nanometer to millimeter and more. In combination with microscopes and high-speed imaging, microfluidic devices allow real time observation of filtration processes. In this review we will give a general introduction on microfluidic devices used to study membrane filtration behavior, followed by a discussion of how microfluidic devices can be used to understand current challenges. We will then discuss how increased knowledge on fundamental aspects of membrane filtration can help optimize existing processes, before wrapping up with an outlook on future prospects on the use of microfluidics within the field of membrane separation.

## 1. Introduction

Membrane filtration is used for a wide variety of applications due to the availability of an abundance of membranes targeted at different separations [1]. The versatility of membranes and membrane modules allows for their application in many different fields, ranging from water purification [2,3] and blood dialysis [4,5] to fermentation broth purification [6] and milk fractionation [7]. For some applications such as water purification, membranes and membrane modules are produced and used on a large scale [8,9]. For other applications, such as dehydration of bioethanol from fermentation broths, scale up is still a challenge due to the high complexity of the process leading to the need to use hybrid systems such as a pervaporation-distillation hybrids [10]. In all membrane processes there are challenges with which researchers and engineers must deal. One of the most researched and reported challenge is the process of flux decline due to concentration polarization and membrane fouling [11,12]. More practical issues are the mechanical and chemical stability, and costs related to production, running and cleaning the membrane modules, which is also directly linked to energy consumption and general environmental impact [13]. These parameters are expected to become increasingly relevant in the coming years and lead to the development of even more sustainable processes.

Membrane separation is mostly considered a mild processing technique since it uses low processing temperatures and low applied pressures (microfiltration and ultrafiltration). For these reasons it is expected to contribute to current world challenges, mainly those related to high energy consumption. For example, the higher demand for food products [14] can benefit from mildly resourced raw materials, leading to more efficient use by purification and isolation of substances of interest and commercial value [6,15,16]. Obviously, this can only be achieved by tuning membranes and membrane processes to the desired application. This may seem very straightforward, but at the same time, doing this repeatedly for every feed stream of interest is not efficient. Especially when keeping in mind that many membranes and membrane processes revolve around similar effects such as fluid mechanics, the understanding of the underlying mechanisms of membrane failure and solute behavior during filtration would greatly speed up process design, and lead to substantial improvements in membrane filtration.

The effects that are ruling flux decrease generally occur at a very small size, short time scales, and in modules that are not that accessible for detailed observations. Computer simulations have been instrumental in achieving better insights, and developing reliable membrane processes; however, validation is always difficult, and for these, microfluidic devices may hold the key. Microfluidic devices have proven to be valuable and powerful tools for fundamental research in many fields such as fluid mechanics [17,18], soft matter [19,20], and biology [21,22] and are currently also starting their way into membrane research. Some of their advantages are their relative low cost, flexibility of design, and the possibility of coupling it with microscopy/high speed imaging techniques [23]. A disadvantage of this approach is the current resolution limitation on the techniques for microfluidic device fabrication. This results in the availability of devices with channels that are much wider than the pores of “real” microfiltration and ultrafiltration membranes; however, these devices still allow for very insightful and interesting experiments and results that can certainly contribute to the advancement of the membrane separation field. We expect these limitations to be overcome in the future as will be discussed later in the outlook session.

**Figure 1:**
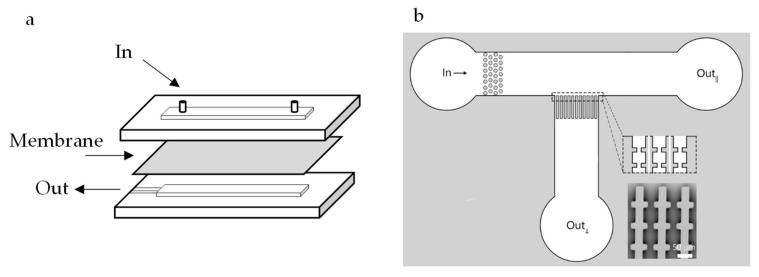
(**a**) Microfluidic configuration with an embedded membrane. (**b**) Microfluidic configuration with an array of parallel channels with the objective to mimic a membrane in an ideal situation. The image has been cropped and modified from the original work [32] and reproduced under the Creative Commons Attribution 4.0 International License [33].

**Figure 2:**
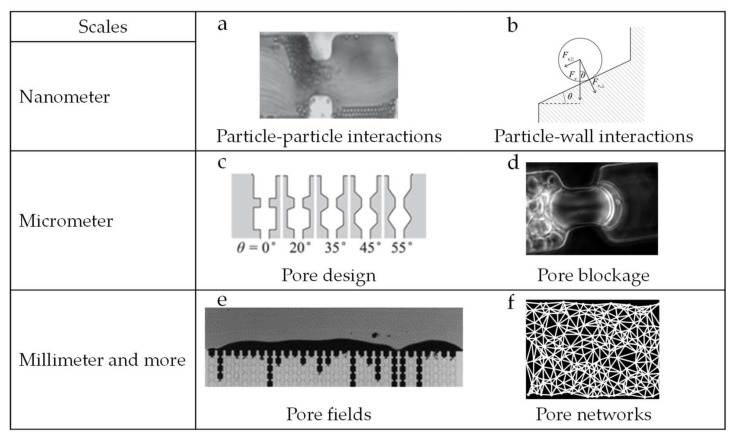
Examples of how microfluidic tools are applied to investigate mechanisms occurring at various scales. At nanometer scale particle–particle (**a**) and particle–wall (**b**) interactions can be studied [41]. Pore design (**c**) [41] and pore blockage (**d**) [42] are topics that can be investigated at micrometer scale, and finally at millimeter scale pore fields (**e**) [32] and pore networks (**f**) [43] have been used to study the collective behavior of particles in porous media. Images a-e have been cropped from their original work and reproduced under the Creative Commons Attribution 4.0 International License [33]. Image f has been reproduced with permission from the author.

In the membrane separation field, several configurations of microfluidic devices have been reported. Microfluidic devices with embedded membranes (see Figure 1a) have been described for particle sorting [24], membrane fouling [25,26], and to study flow in membrane microreactors [27]. In these studies, microfluidic devices are used to support small pieces of membranes and allow for in situ observation of the filtration process. Alternatively, microfluidic devices can be designed as model membrane systems [28,29,30] (see Figure 1b), containing channels that mimic, for example, membrane pores under highly ideal conditions. In this way, microfluidic devices can be used to study and understand phenomena occurring at different scales [31], ranging from nanometers to even millimeters and more (see Figure 2 for illustrative examples).

At the nanometer scale, the study of colloidal particle–particle interaction during flow and particle surface interactions are some examples of topics that have been studied [32]. Material surface modification and functionalization is also part of these investigations, and geared toward minimizing the interactions occurring at nanometer scale [4] (Figure 2 top line). At micrometer scale, idealized membrane mimicking microfluidic devices have been proposed to investigate, for instance, pore geometry in relation to blocking and process optimization, using both hard and deformable particles [28,29,30] (Figure 2, middle line). Finally, at millimeter scale, microfluidic devices have been useful for investigating phenomena related to collective particle behavior, such as surface deposition/cake formation and the (collective) movement of particles in flow [26,34,35] (Figure 2, bottom line). It is good to mention that insights obtained with microfluidics in other fields for example, to study flow [36,37,38] and to separate individual cells [39,40] are very relevant for membrane separation.

A number of reviews have been written on membranes and microfluidics [44,45,46,47]. Some of them describe the applications of microfluidic chips with integrated membranes as used for cell and protein research and gas detection [44,45]. In their review, published in 2006, De Jong et al. [45] discussed the integration of membrane functionality into microfluidic chips. They stated that bridging the fields of microfluidics and membranes can be beneficial for both fields of study. Indeed, in the last years, the use of microfluidic devices to study membrane filtration phenomena has flourished, although the focus has been mostly on microchip fabrication techniques as reviewed in [46,47], and not much effort has gone into bringing the findings that were obtained toward improvement of membrane processes. Therefore, we chose this as the topic of our review.

The current review contains four parts. The first one is a general introduction on microfluidic tools used for membrane filtration related research, covering many variables ranging from device structure to the choice of model particles. The second section covers current microfluidic tools and their use to investigate typical phenomena related to membrane filtration, such as flux decline, and cake layer formation. In the third section, we give an outlook on how we think these insights can be used to optimize current filtration processes. We wrap up with a section in which we present expected future developments, highlighting the role that the additional information obtained through microfluidic investigations can play in terms of development of innovative membrane fractionation processes that allow better use of all components present in raw materials (thus contributing to a more sustainable circular economy).

## 2. Microfluidic Devices as Tools

### 2.1. Structure

As indicated previously, microfluidic devices used to investigate membrane filtration can be separated in two broad categories: microfluidic devices with embedded membranes [4,26,27,48] and microfluidic devices that mimic membrane structures [28,29,30]. As the name already states, devices with embedded membranes are microfluidic devices designed to accommodate a small piece of a membrane in their structure. The advantage of this configuration is that a real membrane is being used, so if measurements of flux and selectivity are important for the study, this might be a good option. However, in situ observation possibilities in these devices can be limited, as is also the case in larger scale observation techniques that monitor cake layer buildup, droplet deposition or sorption of macromolecules (with fluorescent probes) for instance [12,49]. Membrane mimicking devices have designs that reproduce, in an ideal way, the structure of a membrane. Most of these devices contain an array of parallel channels or structures, that would represent the pores in a real membrane. The advantage of this approach is the possibility of observation of particle behavior at individual level. Additionally, the observation of cake layer structure and pore blocking mechanisms in these systems is facilitated through sideways 2D observation. Due to the flexibility of design of microfluidic devices, the structures mimicking a membrane can be fabricated practically at will. Parallel straight through channels [50], parallel channels with constrictions [51], round pillars [52,53] and non-aligned squares [29] are some examples of structures used to simulate membrane filtration with microfluidic devices. Most membrane mimicking microfluidic devices, including the ones cited before, are made of Polydimethylsiloxane (PDMS) via soft lithography. However, new fabrication techniques are being developed such as the fabrication of polyethylene glycol (PEG)-based hydrogel membranes via photo-patterning. These membranes can have their permeability easily controlled with pore sizes closer to those present in “real” membranes, are pressure-resistant and have been reported to be used for the study of microfiltration and ultrafiltration processes [54,55].

**Figure 3:**
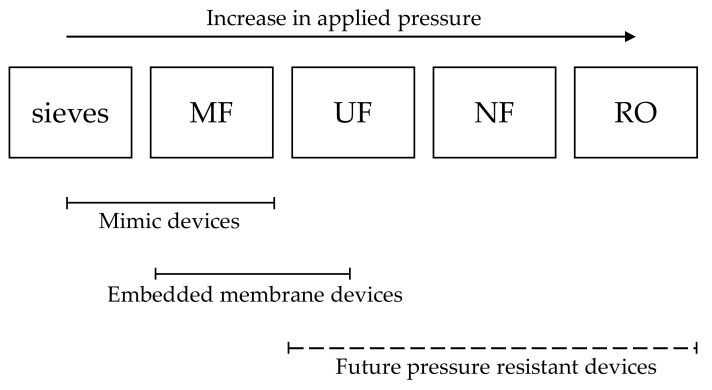
Scheme showing which type of microfluidic devices are currently suitable for the study of different porous media—sieves, microfiltration (MF), ultrafiltration (UF), nanofiltration (NF), and reverse osmosis (RO).

Although microfluidics can be a highly ideal system to study membrane filtration, the results and insights obtained can be of great interest for real membrane processes, mainly microfiltration. Microfiltration is the membrane process that can be most easily investigated since the current available technology allows the production of devices that have pore/channels sizes that are the closest to the ones present in microfiltration membranes. Additionally, microfiltration processes target mostly micrometer sized particles, and these are easy to model and observe with simple optical microscopy techniques. Ultrafiltration processes can still be investigated with microfluidic devices but producing mimicking microfluidic devices can be challenge due to the resolution of the current available techniques that does not allow the production of channels in the smaller range. Microfluidic devices with embedded membranes can be easily used instead where a piece of ultrafiltration membrane is attached to a microfluidic system. Visualization of the process when dealing with ultrafiltration processes can also be more challenging but still possible. Direct observation of accumulation of proteins for example, can be achieved by using fluorescence microscopy of tagged proteins. Other filtration processes such as nanofiltration and reverse osmosis are still not eligible for investigation studies with microfluidic devices mainly due to the high pressure required in these processes and also due to the limitations on microscopy techniques that currently do not allow in situ and real time observation of small specimens such as salts and small molecules (Figure 3).

### 2.2. Foulants

The to-be-tested suspension/foulant will influence the outcomes of the experiments greatly, as would also be the case in any membrane separation experiment. While some studies use real suspensions such as blood in their experiments [56,57], most prefer to use model suspensions such as latex beads suspended in water [28,46,58]. Model suspensions contain in general only one solvent and type of particle, and their choice depends on the objective of the experiment. Water is the most used solvent, while the selected suspended particles can be more diverse. If the suspension is used to gain general insights, basically any particle will do, but if the system should somehow reflect the characteristics of a suspension of interest, various particle properties need to be considered: soft or hard, how large, monodisperse or polydisperse, etc.

The choice between soft or hard model particles depends on either the system that is being modelled or the specific characteristics that the study is aiming to assess. Hard particles are suitable, e.g., for studies aiming at modelling filtration behavior in general since the particle size remains constant during the process, thereby limiting the number of variables to be considered (such as compaction and deformation). Soft particles are mostly chosen as models for cells and protein aggregates, and often microgels are used due to their ease of production, and tunable properties. Soft particles are able to modify size and shape during filtration, therefore making their filtration behavior much more complex [19,59], e.g., leading to added flow resistance when pressurized in a filtration cake layer [60].

The size of the particles will also influence the outcome of the experiments and are ideally as close as possible to the final application. For example, the migration of particles in flow highly depends on their size, and thus also the build-up of concentration polarization and cake layer [61,62]. If colloidal interactions during the filtration process are of interest, smaller particles should be preferred; however, in various cases large particles can also show the behavior of interest making observation much easier. The current technological constraints regarding microfluidic device fabrication should also be considered when selecting particle size. At the moment, the fabrication of devices mimicking membranes with channels smaller than 5 µm can still be a challenge and for that reason, when observation of individual particle behavior is the main objective of a study, the use larger particles will be more adequate while smaller particles can still be used for collective behavior observations in larger channels. The minimum particle size to be used can also be limited by the resolution of the optical methods used to observe the particles during the experiments.

From the previous point, it also follows that particle size distribution is a parameter to consider since it can largely influence the observations. The use of both monodisperse and polydisperse particle size distributions for experiments in microfluidic devices have been reported in the literature [34,35,63]. For experiments focusing on process modelling, monodispersed distributions are preferred since they will not bring extra complexity to the system. However, the use of polydisperse model particles brings model systems closer to feed streams as used in industrial applications, and that will contain particles with a wide range of sizes.

## 3. Understanding Current Challenges in Membrane Processes

### 3.1. Flux Loss/Decrease

Flux decrease and membrane failure are some of the biggest challenges in membrane filtration; concentration polarization, pore blocking, and membrane fouling being the main culprits [7,64]. Many books and reports have described how concentration polarization occurs [6,11,48,64]. In brief, concentration polarization is the build-up of a solute concentration gradient between the membrane and the bulk feed. This happens due to the membrane removing solvent constantly, therefore increasing the solute concentration close to the membrane surface. This added resistance impacts the flux and efficiency of the process negatively. Its effects are mostly minimized by applying cross flow filtration (instead of dead end) in industrial membrane processes.

Concentration polarization may be the onset for cake layer formation if the concentration at the membrane exceeds a certain value due to the growing deposition of particles on the surface of the membrane. The presence of a cake layer affects not only the flux through the membrane, but can also influences the selectivity of the process, either by (partly) blocking pores, or acting as an in situ produced membrane on top of the actual membrane. Reversing the flux (back pulsing, at very high frequency) is a solution that is used in commercial membrane processes to temporarily mitigate these effects, as will be discussed later [64,65]. Ultimately, cleaning liquids will be used to remove the cake layer and ideally any components that might have adsorbed to the membrane surface.

The effect that components have can be very local, on the level of individual pores. This is the so-called pore blocking, which can cause flux decrease and membrane failure. As with cake layer formation, it is possible to reverse the phenomenon (partially) by using back-pulsing and cleaning procedures [7,64]. However, understanding and minimizing its occurrence is preferred, as would be the case for concentration polarization and cake layer formation.

The first studies aimed at monitoring flux decrease and membrane failure were performed by measuring changes in process parameters such as flux and pressure drop. This indirect approach was valuable and brought a lot of insights in flux and pressure dependency, although the obtained results were very case specific and only limitedly valid. Later in time, techniques allowing in situ monitoring of what was happening on the surface of the membrane arose, leading to methods to follow cake formation in real time while still measuring changes in e.g., flux. Some of the techniques used to assess concentration polarization and cake formation during membrane filtration are light deflection techniques, magnetic resonance imaging (MRI), and some optical techniques such as direct observation through the membrane (DOTM) [12]. In 2004, Chen et al. [12] discussed these and other in situ characterization techniques in depth in their review. In the last sentence of this publication, the authors mentioned microfluidics as one of the techniques offering vast opportunities for development of in situ monitoring techniques. Sixteen years later, while the techniques mentioned in their review are still very much used, microfluidics have indeed become this very important and widely used tool for membrane studies, as we will discuss in the next sections.

### 3.2. Pore Blocking Mechanisms

**Figure 4:**
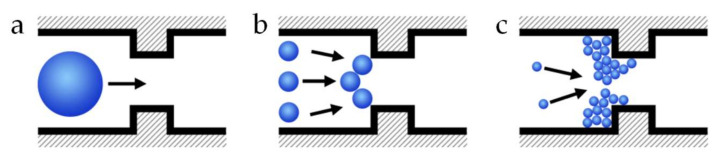
Pore blocking mechanisms: (**a**) sieving, (**b**) bridging, and (**c**) aggregation [43]. Image used with permission from the author.

Pore blocking is the partial or total obstruction of a pore or channel. Partial obstruction happens when particles deposit around the pore entrance, or in the membrane matrix resulting in the constriction of the pore. To elucidate pore blocking mechanisms and the particle–particle and particle–wall interactions that are an important part of this subject, microfluidic devices have been used in the last years. One of the first reports on the use of microfluidic devices as model porous media containing an array of parallel channels to study mechanisms for clogging of microchannels was written by Wyss et al. [66] in 2006. Since then, this approach has gained popularity for the study of clogging of microchannels. Dressaire et al. [23] wrote an extensive review on pore blocking mechanisms in constricted microchannels. They described sieving, bridging and aggregation of particles as the main blocking mechanisms. A pore is blocked by sieving when a particle larger than the opening blocks it based on size exclusion. Bridging occurs when particles smaller than the opening of the pore form an arch of particles across the width of the opening. Finally, particle aggregation promotes pore blocking by the successive deposition of particles in the channel (Figure 4). Mainly for the particle aggregation mechanism, particle–particle, and particle–wall interactions play an important role as described in the theory detailed in their review.

Van de Laar et al. [41] used membrane mimicking microfluidic devices in dead-end mode to study the influence of pore geometry (channel entrance angle) and particle interactions on the clogging behavior of polystyrene particles. They found that at steeper entrance angles, clogging time decreases by almost a factor of 4 as compared to more shallow angles. The authors used particle image velocimetry (PIV) to explore their hypothesis that the effect of the shape of the entrance of the channel on the local flow fields could be responsible for the relationship between failure rate and pore geometry. They, however, found no deviations from laminar flow explaining that the changes on local flow fields were not responsible for the dependence of failure rate on the angle and shape of the pores. Although PIV could not help the authors of this work to explain their results, this valuable technique has been used in many studies on the flow of particles in porous media [67,68,69]. To finally explain their results, a model based on transition-state theory was derived. The model considered the effect of viscous forces on the particle accumulation rate on the walls of the channels. The model also provided the possibility to predict the effect of particle interaction on the clogging rate based on the potential energy of interaction between particles and between particles and wall. They describe this potential energy of interaction as being composed of two terms: An attractive term due to van der Walls interactions at short distances and a repulsive term due to steric repulsions induced by the surfactant. Before aggregation occurs, this characteristic energy barrier, that results from the combination of the two terms, should be crossed. The authors found excellent agreement between their experimental data and theory.

In subsequent work, van Zwieten et al. [32] used microfluidic devices with similar channel configuration to study the kinetics and mechanisms of clogging in crossflow filtration mode. They found that crossflow does not have an influence on the primary clogging rate, but it alters the communication between pores/channels, resulting in a transition from correlated to uncorrelated pore clogging. To explain their findings, they derived a dimensionless number that rationalizes the primary clogging rate and the rate of cake build-up taking into consideration parameters such as the sticking probability, intrinsically related to the presence of attraction forces between the particles.

Pore blocking is mostly the first phenomenon that happens in fouling and membrane failure. Pore blocking can lead to more deposition and cake formation, as is discussed next.

### 3.3. Deposit Layer Formation/Cake formation/Kinetics of Deposition

**Figure 5:**
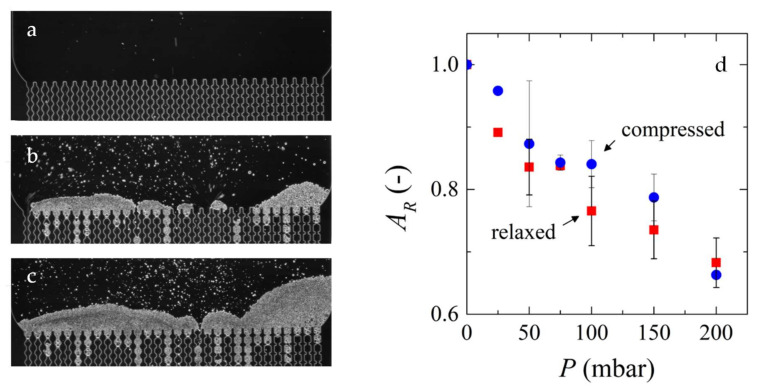
Microscopy images of a deposit layer formation on top of a membrane mimic microfluidic device (**a**) before clogging, and (**b**) in the middle and (**c**) end of the filtration process. (**d**) variation of relative cake area with varying pressure for filtration cakes being compressed (blue circles) and relaxed (red squares) [30]. Image d) has been reproduced under the Creative Commons Attribution 4.0 International License [33].

In one of our previous works [30], we used a membrane mimicking microfluidic device to filter a suspension of micrometer sized microgels (Figure 5a−c). The microfluidic channels were clogged and the process was run until a cake layer was formed on the surface of the channels. The applied pressure to the system was varied in order to determine the reversibility of the cake layer compression and monitored the relative area of the cakes in the microscopy images after pressure was applied to the system (Figure 5d). We found that the particle deposits can be reversibly compressed if there is availability of solvent for reswelling due to capillary forces, which indicates that we are looking at the thermodynamic state. In another work [20], we compressed the same microgels under different pressures and observed their changes in volume and shape for individual particles. We saw that the microgels deswell and deform, forming facets under all conditions analyzed. However, the extent of response of both mechanisms to pressure varied greatly. At low applied pressures, deformation is the predominant mechanism, while that is deswelling at higher pressures.

Understanding the patterns of particle deformation is important since they can influence cake layer properties, and thus the flux that will be obtained in an actual membrane filtration experiment. Linkhorst et al. [19] aimed to correlate permeation performance to filter cake morphology based on deformation of single microgel particles. They used a membrane mimicking microfluidic tool to build amorphous and crystalline filter cakes. The shapes of deformed particles were dependent on the type of cake layer, which in turn affected the degree of deformation, and thus influenced cake permeability through the resulting pore size. Using a similar system, Linkhorst et al. [28] followed the translocation and interaction of individual particles in the filter cake.

Other factors can also influence cake formation and behavior such as suspension ionic strength and pH [70,71], feed concentration [71], and particle size distribution [34,35,63]. In Di et al. [71], fluorescent polystyrene latex particle (0.4 µm) deposition was evaluated. Regarding the pH, they found that at higher values, the deposition was homogeneous and its thickness equivalent to a monolayer of particles. At lower pH values, the deposition was heterogeneous with a higher deposition volume in comparison with deposition at higher pH. At lower pH, particle aggregation was favored followed by deposition on the membrane. When considering ionic strength, they found that at low ionic strength, the deposition was homogeneous due to attractive forces between the particles and the membrane. Increasing the ionic strength changed the membrane surface charge, therefore creating repulsive forces, while at the same time promoting particle aggregation, overall resulting in heterogeneous deposition.

In the work of Di et al. [71] the influence of feed particle concentration was studied, but they did not find a strong influence on deposition behavior, unlike others. Ngene et al. [34] used microfluidics to study the deposition of polystyrene particles of two sizes and varied the composition of the suspension. They determined the cake thickness and cake porosity and found that the cake thickness increased with increasing fraction of larger particles (as expected), which is in line with modelling studies of Kromkamp et al. [72]. Cake porosity also varied with the fraction of larger particles, with a minimum at 0.5.

### 3.4. Flux Decrease Mittigating Measures

In practice, membrane fouling is minimized using backwashing, also known as back-pulsing, back-flushing, or back-shocking. During this procedure, the flow from feed-permeate is reversed to permeate-feed, to remove particles that may have deposited on the feed side of the membrane [64,73]. Microfluidic devices, mainly membrane mimicking systems, have been used in the last years to study these processes using a range of designs.

Lohaus et al. [65] combined microfluidic experiments with numerical simulations to visualize and describe collective particle dynamics during backwash. For the microfluidic experiments they used monodisperse polystyrene suspensions (4.2 µm diameter) that were flown through the microfluidic chip, and a deposition layer was allowed to form before the backwashing experiments started. They found that backwash efficiency was controlled by particle clusters, showing that single-particle models were not representative of the processes occurring during backwash. Simulation results showed two main events during backwashing: partial resuspension of particle clusters and orientation of attached particle clusters toward the region of lower drag. Furthermore, simulation results revealed that particle membrane interactions influenced backwashing much more strongly than particle interactions.

**Figure 6:**
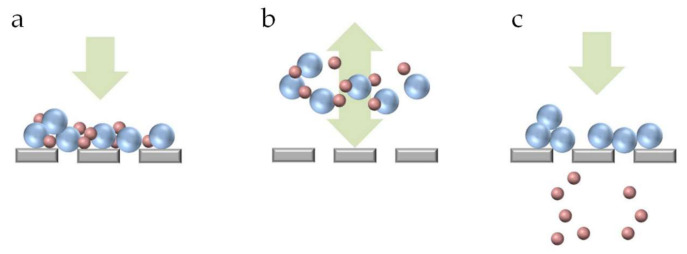
(**a**) Particle deposition on top of the filter. (**b**) Particles moving away from the filter as a result of the in-phase vibration (fluid oscillation). (**c**) As a result of the disturbance of the deposition layer, smaller non-target particles pass through the filter [74]. Image reproduced under the Creative Commons Attribution 4.0 International License [33].

Microfluidic filtration devices coupled with pressure oscillators have been reported to generate a periodic back and forth streaming [74] that can be considered analogous to backwashing in membrane filtration. Yoon et al. [74] used such combination to separate a bidisperse suspension containing polystyrene particles with 5 µm and 20 µm diameter. Their objective was to have the smaller particles go through the pores while retaining the larger particles, but they observed that many smaller particles got trapped between the larger particles in the deposit layer on top of the pores, rendering them unavailable for separation. The reversed flow disrupted the large particle deposits, therefore releasing the smaller particles, and thus allowing further separation (Figure 6). Although not directly aimed at this, this last study was also instrumental for understanding and optimizing membrane cleaning processes.

### 3.5. Biofilms

Failure due to biofouling is another challenge of membrane filtration processes, especially in membranes used in bioreactors. Proteins, carbohydrates, cells, and biofilms are some examples of biofoulants [75,76]. Most foulants can be removed or have their quantity reduced using pretreatments in the feed stream. However, biofilms are composed of microorganisms that are able to multiply, and produce excretion products known as extracellular polymeric substances (EPS), that protect them from different stressors such as antibiotics and hydrodynamic shear [77]. For this reason, if not 100% of the cells are removed, there will still be enough cells to multiply using the resources present in the feed stream and recolonize the biofilm [75,78,79,80].

The aforementioned reasons clearly illustrate the extensive effects that biofilms can have, making them an interesting subject of research also in combination with microfluidic devices. Microfluidics allow the direct observation of the biofilm layer, and in their review, Pousti et al. [81] highlighted the use of microfluidics coupled with various techniques, such as microscopy, electrochemical measurements, spectroscopy, and chemical imaging. In another review, Karimi et al. [77] discussed the relevant physical processes in biofilm formation such as propulsion mechanisms and hydrodynamics effects. They also discussed some microfluidic techniques used to study these mechanisms, which also included the formation of biofilm streamers, filamentous structures formed when flowing microorganisms in confined systems. These can be found in membrane systems, in between spacers in nanofiltration and reverse osmosis, leading to decreased process efficiency [82].

Marty et al. [82] studied the formation of streamers and more specifically, the effect of channel size, channel connectivity and tortuosity, and the effect of flow rate, using membrane mimicking microfluidic devices and bacterial suspensions. They found that increasing channel size decreased the average streamer length and their formation rate. Increasing channel tortuosity and flow rate had the opposite effect, both increasing their average streamer length and enhancing formation.

Rusconi et al. [83] used microfluidic devices together with numerical simulations to understand the effects of hydrodynamics and complex biofilm structure formation such as streamers. Their devices allowed observation of streamer formation in curved channels under a laminar flow. They found that biofilm streamers were likely to connect to the microchannel walls in the corners of the channels, where vorticial motions were detected. Their results showed that hydrodynamics affect bacterial streamer formation, which is expected to be much more widespread than anticipated.

Sendekie et al. [84] used a membrane mimicking microfluidic device composed of an array of 5 µm wide channels to investigate the interplay between biological particles and colloidal particles at a constriction. For that they used monodisperse latex particles and fluorescent *Escherichia coli* in mixed suspensions. They observed particle deposits in the upstream zone of the device and formation of streamers in the downstream zone. They found that the presence of *E. coli* delayed clogging in comparison to scenarios where only latex particles were present. They hypothesized that this difference may be due to the slippery bonds inside the particle/bacteria aggregates.

As can be seen, the behavior of bacteria and biofilms is complex with still many unknowns. However, it is clear that microfluidic devices are a great tool for clarification.

## 4. Optimization of Existing Membrane Processes

In the previous sections we discussed how microfluidic tools can be used to study and understand phenomena that occur during membrane filtration. In this section, we will discuss how similar tools can be used to optimize existing membrane processes parameters such as flux and selectivity. We also comment on how membrane pore design can be improved.

### 4.1. Improving Flux and Selectivity

Flux and selectivity are the main parameters in membrane processes, and improvement of flux and mitigation of fouling go hand in hand. In this regard, microfluidic devices can be used to investigate a variety of possibilities, and when used in combination with fluid mechanics and modelling, very detailed insights in membrane filtration can be achieved including possibilities for improvement of selectivity. The ultimate aim is increased efficiency in purification of target molecules/particles as well as increased yield in the recovery thereof. Many studies have been reported on separation and detection of particles, ranging from nanoparticles to cells.

Sauret et al. [85] developed a microfluidic chip to detect contaminants in colloidal suspensions. Their aim was to produce a device that would be clogged by the contaminants, thereby allowing their quantification. For their overall approach to be successful, the authors had to ensure separation based on size exclusion phenomena. They observed that the shape of the contaminant is a factor that influences clogging behavior greatly.

Separation processes based on inertia effects in microfluidics have recently been reviewed by Dijkshoorn et al. [86]. Using inertial lift, separation of particles of different shape has been reported by [87,88]. Because of this principle, particles can migrate to dynamic equilibrium positions away from the membrane, assuming different positions across the fluid streamlines depending on their shape. The separation of microalgae cells [87,88] and blood cells [22,89] are examples of separations shown to be achievable with this kind of approach.

**Figure 7:**
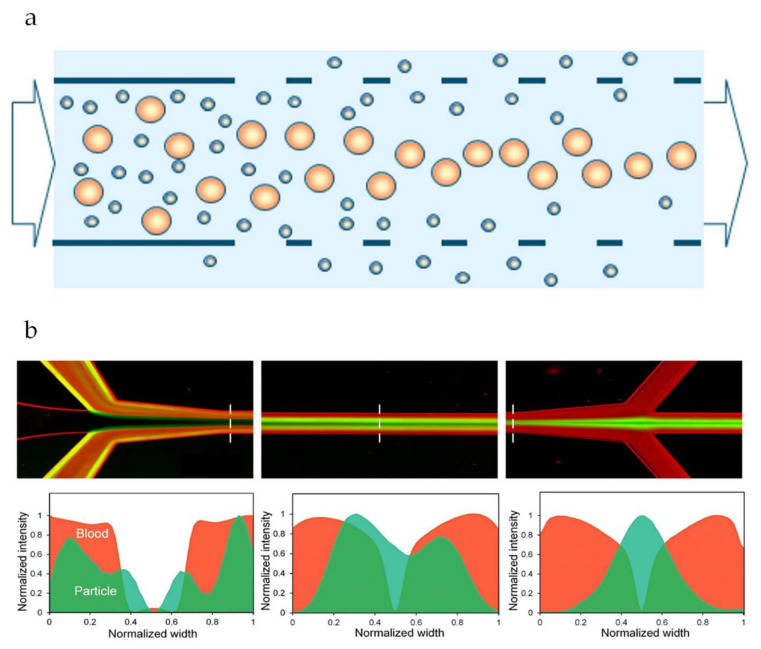
(**a**) Schematic representation of shear induced diffusion separation in a porous channel [14]. (**b**) Top: Merged images (fluorescent+inverted bright field) demonstrating the concept of shear induced diffusion separation of a mixture of blood (red) and PS particles (green). Bottom: Intensity profiles of blood and particle streams across the dashed lines present on the merged microscopy images [93]. The images have been cropped from their original work and reproduced under the Creative Commons Attribution 4.0 International License [33].

Shear induced diffusion is another inertia based particle migration mechanism that has also been reported as a way to manipulate and mitigate particle deposition, and improve flux and retention greatly [61]. Due to collective particle interaction behavior, a mixture of particles flowing through a channel will automatically segregate according to size, with large particles migrating faster than small ones. Consequently, larger particles will tend to move away from the walls of the channels while smaller particles will stay closer to the walls (see Figure 7a). It is true that separation using microfluidic devices and membrane separation processes such as microfiltration rely on different principles. For this reason, inertia based microfluidic separation shows potential as an alternative for membrane separation processes for certain applications [86]. Shear induced diffusion has been studied in the last years as a possible base for innovative membrane technology that does not suffer from particle accumulation and flux decrease, and can be carried out at low energy demand [61,90] compared to classic microfiltration (~30%).

Microfluidic devices have been a very important tool for shear induced diffusion research [90]. So far, most reports on the use of microfluidic devices to study shear induced diffusion for separation purposes use either model particles [91,92] or blood [93] (Figure 7b). These applications have shown the efficiency of the technique and its potential for future applications. Furthermore, the principle has been shown to hold when using well-defined metal sieves for the separation of latex particles, algae, yeast, and emulsion droplets in a purpose-built module consisting of a closed area for particle migration to take place, and a porous area to allow for separation to occur [14,61].

Dow et al. [94] used microfluidic devices for the acoustic separation (acoustophoresis) of bacteria from blood, basically using an additional driving force in combination with a microfluidic device. In this technique, the microchannel acted as a resonant cavity. The resulting wave of pressure exerted forces on the suspended particles according to size, density, and compressibility. The application of these different forces allowed the separation of particles, as is also used on larger scale, e.g., for cell removal from fermentations. This approach was also described by Fornell et al. [95] for the separation of a binary mixture of polydimethylsiloxane (PDMS) and polystyrene particles.

### 4.2. Surface Modification

Membrane surface modification is often directed toward increased hydrophilicity resulting in improved resistance against fouling [96], and consequently membrane selectivity. However, the actual effects that are created are not that well understood. For dialysis, surface modifications are mostly related to the improvement of membrane selectivity since it is very important to selectively reject target molecules in the blood. Since the surface properties of microfluidic devices can also be modified, depending on the construction material of course, microfluidic devices could be truly helpful in creating in depth insights in the effects created by surface modification. The use of surface functionalization to minimize fouling was reported by Ausri et al. [4] for microfluidic devices developed for hemodialysis. Their device was composed of two PDMS chambers and a cellulose ester (CE) membrane. Surface fouling in the microfluidic device and membrane obstructed the flow decreasing the efficiency of the toxin removal from blood and even influencing selectivity. Both the PDMS device and the CE membrane surfaces were grafted with polyethylene glycol (PEG) with the PEG polymer resisting protein binding, and decreasing fouling.

In their recent review, Gerami et al. [47] discussed the fabrication and application of microfluidic chips to study the transport processes in applications such as complex hydrogeological systems and petroleum production. They also focused on a variety of works where the surface functionalization of the microfluidic chips played an important role in achieving their objective: improved oil removal.

Bi et al. [97] reported the modification of poly(methyl methacrylate) (PMMA) microfluidic chips for biomimetic surfaces with reduced biofouling. The microfluidic chip, designed to be used in electrophoresis of proteins, was functionalized with 2-methacryloyloxyethyl phosphorylcholine (MPC), turning the surface of the chip hydrophilic and biofouling resistant.

Different materials have been used to fabricate microfluidic devices such as glass, silicon, PDMS and PMMA. The choice of material depends on the desired application, but all mentioned materials can have their surface modified. The method used varies: silanization is a method widely used for inorganic materials such as glass and silicon. Polymeric materials such as PDMS and PMMA can readily be modified by plasma oxidation, polyelectrolyte multilayer coating, and laser radiation [67].

### 4.3. Pore Design

In general, membrane processes are developed using the average pore size of the membrane as a guide. This approach has led to many successful large-scale applications. However, given the advances in membrane fabrication, and increased control over pore design using for instance clean room facilities, the development of the membrane processes of the future could be done at a higher level of refinement level [98].

Studies focusing on the effects of microfluidic channel structure such as tortuosity and shape have been reported [29,52,98,99]. Channel tortuosity has been investigated by Bacchin et al. [29] and Maitri et al. [52]. Bacchin et al. used membrane mimicking microfluidic devices presenting non-aligned square pillars instead of an array of parallel channels to simulate the effects of tortuosity and connectivity of pores. With the device, they filtered a suspension of latex particles and observed clogging behavior that started to take place in the internal spaces first and progressed upstream, blocking the channel entrances. A similar phenomenon can be observed in microfluidic devices containing constricted channels. Whenever clogs happen deeper in the channels, a filtration cake forms on top of the clog and inside the pores, eventually spilling out of the pore [30,32]. Maitri et al. also used microfluidic devices containing pillars, to assess the effect of the organization of the pillars: either ordered or random. They flowed polystyrene beads through the channels and found that flow behavior in the device with ordered pillars was ordered and predictable; whereas the opposite was found when random pillars were used. In practice, the structures found in membranes are mostly random, and the study of Maitri et al. clearly pointed out that knowledge on ideal systems needs to be extended to be useful for real applications.

The effects of pore connectivity and pore network structure were also studied by Van de Laar [43]. He used microfluidic devices containing a disordered network of connected channels to evaluate the effect of these structures on the displacement of oil by water. The channels were initially filled with oil and water was flown with the objective to displace the oil. The angle of the channels in relation to the major flow axis showed to have an influence on the efficiency of oil displacement. Initially, the flow of water displacing the oil happens mostly through channels with angles lower than 45°. When breakthrough occurred, a second stage began where channels with higher angles started filling up with water. However, the second stage happened in a much slower timescale in comparison with the first stage. Additionally, the total systems were never used: even at the end of the second stage, many channels were left unused. His results are particularly interesting for the understanding and optimization of oil recovery processes but are expected to be also very insightful regarding cleaning processes during which also one liquid needs to be replaced by another. If similar effects occur, this implies that cleaning processes do not only take very long (which is the case) but can also not be effective in the sense that all channels are cleaned.

The effect of channel shape has been studied by Massenburg et al. [99]. They used a membrane mimicking microfluidic device composed of an array of tapered channels. They varied channel mouth width and length and found that increasing the mouth width of the channel or decreasing its length leads to a decrease in hydrodynamic resistance of the channel. This happened because of the higher shear stresses near the constrictions, therefore preventing the attachment of particles to the channel walls. Their results are of importance to systems where channels become narrower, as in blood vessels.

### 4.4. Determination of Particle Properties (Auxiliary Techniques)

Studies that have been carried out in other fields with microfluidic tools can indirectly contribute to the advancement and understanding of membrane processes in general. Particle properties play an important role in the behavior during filtration processes, and novel methods to determine the compression and deformation behavior [19,42] and the elastic modulus [100] of soft microgels has been suggested in other science fields.

Capello et al. [100] determined the Poisson’s ratio of polyethylene glycol (PEG) hydrogel particles. They presented a simple technique that allowed in situ measurement of the swollen particles, without the need to dry them. After fabrication, the soft particles went through a constriction smaller than their width, promoting deformation. The particle was then allowed to reach equilibrium and the variation in shape of the particle in equilibrium was used for the calculation of the Poisson’s ratio. The authors only took particle deformation into account and did not mention if their particles experienced any variation in size while reaching equilibrium inside of the constriction, but the fact that the Poisson’s ratio could be estimated was an important step forward.

In one of our previous works mentioned earlier [20], we demonstrated that microgels can deform and deswell simultaneously when subjected to isotropic forces. In a follow-up work [42], we observed deformation of individual microgels going through microfluidic constrictions. We quantified the variations of shape and volume of the 2D projections of the particles, and found qualitative agreement with previous work that did not involve microfluidics [20].

## 5. Outlook

One of the challenges of using microfluidic devices is to make sure that the results obtained in these highly ideal models can be translated to real life membrane applications, in this way improving the processes and solving currently faced issues.

Advances in microfluidic device fabrication over the last few years have made the production of microfluidic devices with higher complexities possible and more affordable, decreasing the gap between model systems and real-life applications. New fabrication techniques such as the before mentioned devices with photo-patterned hydrogel membranes [54] and 3D printing are increasingly being used for the fabrication of devices with higher complexities and resolution. 3D printing is also bringing improvements to classic fabrication techniques such as soft lithography. The fabrication of more robust devices and complex flow regulation components can be achieved with 3D printing in combination with devices fabricated with soft lithography [101].

The possibility of fabricating microfluidic devices with higher complexities will also broaden the possibilities in terms of applications and we expect that the scale in which things can be observed and investigated is not going to be a limiting factor for long [67]. The use of microfluidic devices with higher complexities could also help develop new separation methods based on, for example, hybrid and discontinuous membranes [102,103].

On the other hand, research on isoporous membranes and microsieves are advancing [104,105,106,107] and the practical use of these systems will mean that results obtained with microfluidic devices could be more relatable, since these systems present inherently lower complexities. In this case, the advancements of membranes and microfluidic model systems could meet halfway. A simulation work by Brans et al., showed clearly how full/partial blocking of pores influences flux, and because of that also retention. They concluded that for fraction processes in which particle size is close, it is better to have full blocking of pores, while for components that are more far apart in size, partial blocking may be preferred [98,107].

This review focused mainly on microfluidic devices with embedded membranes and microfluidic devices designed to mimic membrane filtration. However, as microfluidic devices are very versatile, many studies have been conducted on the use of microfluidic devices for particle and cell separation with designs and principles that do not resemble membranes, such as devices with spiral channels and ratches [21,22,90,108]. In these cases, the devices have been developed as the optimum solution for a certain type of separation. However, they can still bring insights and interesting results that can be applied in the understanding of filtration processes. One of the advantages of using tailor made microfluidic devices for the separation of particles and cells is high selectivity. In the case of separation of cells, microfluidic devices can be considered much gentler in comparison with membrane separation or even centrifugation, that is much more detrimental, thus lowering the loss of cells due to damage. Another advantage is the possibility to follow the separation process closely by using microscopy techniques allowing the operator to adjust process parameters according to the progression of the separation process. The low throughput of individual microfluidic devices makes them suited for specific applications and putting many in parallel may result in higher production and running costs, unless smart designs are found, as reported for e.g., emulsification, and elaborated on next.

Emulsification is another process where microfluidic devices have been increasingly used. Due to their versatility and the possibility to control flow in a very precise way, it is possible to use microfluidic devices to produce monodisperse emulsions as well as more complex emulsions such as multiple emulsions [109,110] what can still not be achieved with membrane emulsification. As in the application of microfluidic devices for separation of particles and cells, the yield of microfluidic emulsification is generally low. Scaling up efforts have been described successfully [111,112], making the future of large scale microfluidic emulsification look bright.

We also expect that the discussed advancements in microfluidic device fabrication will lead to further optimization of microfluidic filtration processes broadening their application on fields such as biomedicine and food safety. Regarding food safety, the use of microfluidic devices as sensors has already been investigated to assess product freshness and the presence of pathogenic microorganisms. This application is not only expected to assure food quality and safety but also avoid food waste by assessing quality and freshness in a more objective way than the well-known and used “best before” date. More detailed discussion on the use of microfluidic devices as sensors for food applications can be found on the reviews by Schroën et al. [14] and Warriner et al. [113]. For biomedical applications, there have been studies on the use of microfluidic device systems for diagnostics [114], cell separation and sorting [39,85], and the development of organs on a chip [115,116]. We expect that the advancement of microfluidic devices production will make their use for these applications common in the future.

## 6. Conclusions

The use of microfluidic devices to study membrane filtration processes in the last years has highlighted their potential as important tools for the optimization of membrane processes. In this review, we discussed how microfluidic devices can be instrumental in identifying phenomena occurring during membrane filtration at different scales. We first gave an overview on the most common configurations used as well as the important parameters that should be taken into account when designing a microfluidic experiment, such as device configuration selection and the selection of the composition of the feed stream. We also discussed scientific works that have used microfluidic devices to study current challenges being faced in membrane filtration such as membrane failure and decrease in efficiency due to particle accumulation and pore blocking. Efforts in using microfluidic devices to optimize membrane processes have also been discussed. Among the topics are the improvement of flux and selectivity, improvements in pore design and surface modification of membranes. Finally, the future of the technology was discussed in the outlook section to bridge insights obtained in highly ideal systems such as microfluidic devices and real-life membrane processes. We highlight innovative separation processes that, for example, make use of shear induced diffusion to mitigate negative effects related to particle deposition and accumulation. For all the reasons mentioned above, we believe that microfluidics have great potential as fundamental research techniques that can be expanded much further and may serve as a basis for innovative processes.
